# Biomarkers Associated With Tumor Ki67 and *Cathepsin L* Gene Expression in Prostate Cancer Patients Participating in a Presurgical Weight Loss Trial

**DOI:** 10.3389/fonc.2020.544201

**Published:** 2020-09-17

**Authors:** Andrew D. Frugé, Kristen S. Smith, Jennifer R. Bail, Soroush Rais-Bahrami, Wendy Demark-Wahnefried

**Affiliations:** ^1^Department of Nutrition, Dietetics and Hospitality Management, Auburn University, Auburn, AL, United States; ^2^Department of Nutrition Sciences, University of Alabama at Birmingham, Birmingham, AL, United States; ^3^Department of Urology, University of Alabama at Birmingham, Birmingham, AL, United States; ^4^Department of Radiology, University of Alabama at Birmingham, Birmingham, AL, United States; ^5^O’Neal Comprehensive Cancer Center at UAB, University of Alabama at Birmingham, Birmingham, AL, United States

**Keywords:** neoplasms, prostate cancer, weight loss, tumor proliferation, gene expression, cathepsin, microbiota

## Abstract

Our previous presurgical weight loss trial among 40 prostate cancer patients found that rapid (but not slow) weight loss resulted in increased tumor Ki67 and *Cathepsin L* (*CTSL*) gene expression. In follow-up analyses, we strove to better understand these unexpected findings. A correlative study was undertaken by performing additional analyses [free fatty acids (FFAs), plasma *CTSL*, and inflammatory cytokines] on remaining pre-post intervention sera and exploring associations with extant data on tumor Ki67, body composition, physical activity (PA), and fecal microbiota. Positive associations were observed between changes in % body fat and FFAs (ρ = 0.428, *p* = 0.026), insulin (ρ = 0.432, *p* = 0.019), and Interleukin-6 (ρ = 0.411, *p* = 0.041). Change in Ki67 was inversely associated with change in lean mass (ρ = −0.912, *p* = 0.001) and change in insulin (ρ = −0.650, *p* = 0.042). Change in insulin was also associated with *CTSL* (ρ = −0.643, *p* = 0.024) and FFAs (ρ = −0.700, *p* = 0.016). Relative abundance of *Bifidobacterium* was associated with *CTSL* (ρ = 0.627, *p* = 0.039) and FFAs (ρ = 0.691, *p* = 0.019); Firmicutes was positively associated with change in PA (ρ = 0.830, *p* = 0.003). Contrary to hypotheses, FFAs decreased with systemic fat loss. Moreover, although glucose metabolism improved, it was inversely associated with Ki67 and *CTSL.* Lean mass loss was highly correlated with increased Ki67. The relationships between prostate tumor Ki67 and *CTSL* and weight loss associated changes in FFAs, lean mass, and fecal microbiota warrant further investigation.

## Introduction

Prostate cancer (PCa) is the most common non-cutaneous malignancy and the second most common cause of cancer-related mortality among men in the United States (1). Obesity is a known risk factor for various cancer types, and affects one-third of men in the United States (2). Although obesity is not associated with the overall risk of PCa, it is implicated in increased risk for aggressive PCa and cancer death (3, 4). Only two studies have been conducted investigating the effects on voluntary weight loss on tumor biology in treatment-naïve PCa patients.

We recently published findings from a randomized controlled trial that explored effects of a weight loss intervention in newly diagnosed PCa patients electing radical prostatectomy as primary treatment (5, 6). We hypothesized that men randomized to the weight loss arm would demonstrate decreased tumor proliferation as assessed by Ki67, a widely accepted biomarker used for pharmacological studies, as it responds to changes in diet and nutritional status (7, 8). However, tumor proliferation increased, rather than decreased with weight loss, and was higher in the intervention group compared to controls at the time of surgery (6). Corroborating our findings, Henning et al. (9) later reported results of a similar trial in which men assigned to weight loss manifested a trend toward higher tumor Ki67 compared to controls.

**FIGURE 1:**
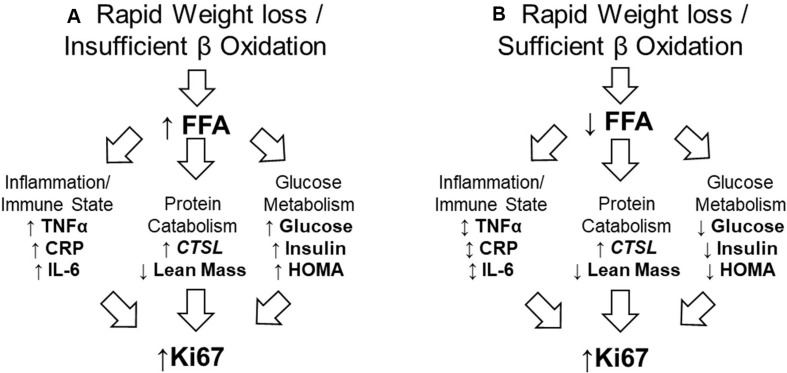
Proposed and observed models of relationship between rapid weight loss and increased tumor proliferation rate. **(A)** The proposed model assumed rapid weight loss would result in insufficient utilization of FFA. This would result in higher serum FFA, which would promote inflammation, protein catabolism, and impair glucose metabolism; all of which work in concert to promote tumor proliferation rate. **(B)** In our sample, FFA were efficiently used, having no effect on systemic inflammatory markers, but promoting protein catabolism while improving glucose metabolism. This suggests the catabolic state alone may have contributed to increased tumor proliferation rate. FFA, free fatty acid; TNFα, tumor necrosis factor-alpha; CRP, C-reactive protein; IL-6, Interleukin-6; *CTSL*, Cathepsin-L; HOMA, homeostatic model assessment (of insulin resistance).

We additionally reported gene expression data, finding that Cathepsin L (*CTSL*) a protease involved in tumor development and metastasis (10, 11), was upregulated in tumor specimens of men in the weight loss group. Recent human trials have indicated that negative energy balance in overweight and obese adults can significantly alter fecal microbiota composition (12) and nutrient absorption (13); differences that are associated with altered glucose metabolism (14). Given the findings of these novel trials and potential for a host-microbiota interaction, we conducted additional biospecimen analyses, hypothesizing that rapid weight loss experienced by our participants would result in increased circulating free fatty acids (FFAs), which would be associated with increased systemic inflammatory markers, dysregulated glucose metabolism, and increased protein catabolism, leading to increased Ki67. Figure 1 is representative of the hypothesized model (Figure 1A) in contrast to the observations based on the data analyzed and presented herein (Figure 1B). The objective of this study was to elucidate mechanisms that may have contributed to our clinical trial findings and may inform future study designs.

## Materials and Methods

Detailed methods of this study have been previously published (5), and are briefly summarized below.

This two-arm, single-blinded, presurgical randomized controlled trial was conducted in overweight and obese men recently diagnosed with PCa. Participants were randomized to either the control group or intervention consisting of a diet and exercise regimen designed to promote a weekly weight loss of ∼1 kg. Primary outcomes of this study were feasibility, based on enrollment, retention, adherence, and safety, while secondary outcomes explored changes in biological measures and tumor markers.

### Eligibility and Study Design

Men were recruited after diagnosis of PCa and were eligible if they had a body mass index (BMI) ≥25 kg/m^2^, had at least 23 days until prostatectomy and were not undergoing any neoadjuvant treatment. This protocol was approved by the University of Alabama at Birmingham Institutional Review Board (Protocol F11051002). Written informed consent was obtained from all participants, baseline measures were obtained, and follow-up measures occurred shortly before prostatectomy. The participants were randomized to immediate or delayed (post-radical prostatectomy recovery) weight loss intervention. In the immediate group, a Registered Dietitian counseled participants twice weekly to consume approximately 1,000 kilocalories below estimated needs. To adhere to guidelines that physical activity (PA) be included as a key component of weight loss interventions to promote negative energy balance, as well as conserve lean body mass (15), an exercise physiologist guided participants through daily aerobic training, increasing intensity and volume as tolerated. Participants were encouraged to exercise two of the days each week under supervision.

### Anthropometric Measures

Height and weight were measured using standard procedures and used to calculate BMI (kg/m^2^). Body composition was assessed via dual X-ray absorptiometry (Lunar GE iDXA, Fairfield, CT, United States).

### Physical Activity

Patient activity levels throughout the study duration were determined using 7-day Physical Activity Recalls administered by trained personnel and were reported as average daily metabolic equivalent (MET) hours (16).

### Circulating Biomarkers

Blood was obtained via venipuncture after a 12-hour fast and analyzed in duplicate. Serum tumor necrosis factor alpha (TNFα), Interleukin-6 (IL-6), high sensitivity C-reactive protein (hsCRP), and insulin as well as plasma *CTSL* and Cathepsin-S (*CTSS*) were analyzed using Meso Scale Diagnostic imaging technology (MSD, Rockville, MD, United States), and serum FFAs were measured on a Sirrus Stanbio analyzer (Boerne, TX, United States) using a Wako reagent (Richmond, VA, United States); analyses were performed according to manufacturer’s directions.

### Gut Microbiome

Stool samples were collected from participants using sterile wipes, which were then placed in a plastic bag and frozen until collection at appointment. Fecal DNA isolation kits were used to isolate microbial DNA. Samples were prepared and polymerase chain reaction (PCR) was used to target the variable region 4 (V4) of the 16S rRNA gene using an Illumina MiSeq (17). Data were analyzed with the Quantitative Insights into Microbial Ecology (QIIME) suite, version 1.7 (18). Sequences were clustered into operational taxonomic units (OTUs) to produce a table with taxonomic identifications and relative abundance data. Based on the relative abundance of OTUs among these samples (19), we selected the top ten phyla and top 25 genera for analyses. Alpha diversity metrics (observed species, Chao1, whole tree phylogeny) were generated and used for correlation studies.

### Tumor Biomarkers

Two pathologists assisted with this investigation; one reviewed clinical pathology reports and all slides for each case, and selected slides with adequate and representative tumor tissue, while the other confirmed the highest PCa tumor grade present. Both pathologists were blinded to study condition. Percent of Ki-67 positive cells were determined from prepared slides at a 1:100 dilution (clone:SP6, Thermo Fisher Scientific, Pittsburg, PA, United States). Intensity levels (0, 1, 2, 3) were multiplied by the percentage staining and divided by 100.

The Nanostring nCounter system (Seattle, WA, United States) was utilized to quantify gene expression within tumor tissue. Cases were selected based on adequate specimen tumors, and six cases from each study arm were analyzed. Cases from the intervention arm, who lost greater than the median amount of weight, were matched based on International Society of Urological Pathology grade grouping system, race, and age (±5 years) to weight-stable controls. Tumors were identified and macrodissected from paraffin-embedded surgical specimens. RNeasy FFPE kits were used for RNA isolation (Qiagen, Valencia, CA, United States). Samples were processed according to manufacturer’s instructions on the Nanostring nCounter Flex system using the GX PanCancer Pathways Panel and the GX PanCancer Immune Profiling Panel. Expression of well-characterized housekeeping genes and spiked-in exogenous positive controls in each sample were used to calculate a normalization factor. This normalization factor was applied to raw counts from nCounter outputs. Cathepsin-L (*CTSL*) was identified previously and these data were used for the current analysis.

### Statistical Analysis

Statistical analyses were conducted in SPSS 25.0 (IBM Corp., Armonk, NY, United States). Descriptive statistics were obtained for all biomarkers and paired *t*-tests were used to determine longitudinal changes for normally distributed anthropometrics and biomarkers. For non-zero Ki67 values <5, an assigned value of 2.5 was used. For plasma *CTSL* values <6.2 ng/mL, an assigned value of 3.1 was used. Wilcoxin signed rank tests were used to determine longitudinal changes for skewed data. Thus, Spearman correlations assessed relationships between changes in weight, body composition, biomarkers, and Ki67. Due to the exploratory nature of this study, correlation studies were not corrected for multiple testing; results with *p* < 0.05 are highlighted.

## Results

### Participant Characteristics

Characteristics of all study participants have been reported previously (6). Twenty-nine participants had adequate pre- and post-intervention anthropometric and biomarker data and were included in this study. Men were 60 ± 7 years old (range, 51–73 years) and weighed 93.9 ± 11.8 kg with BMI 30.1 ± 3.1 kg/m^2^ and 35.4 ± 5.1% body fat at baseline. Roughly one-third of participants reported their race as non-Hispanic Black (31%) and the remainder were non-Hispanic White. All men had Gleason sums of 8 or less at diagnosis. Over the course of the study, these men lost 3.4 ± 4.0 kg body weight and 1.6 ± 1.9% body fat (*p* < 0.0001 for both), and as a whole, did not have significant changes in insulin, FFA, TNFα, IL-6, or hsCRP.

### Tumor Biomarkers Associations

Correlations between changes in tumor proliferation rate and other biomarkers are reported in Table 1. Change in Ki67 was inversely associated with change in lean mass and change in serum insulin. Change in insulin was also inversely associated with *CTSL* and FFA. We then analyzed *CTSL* and *CTSS* in plasma to compare systemic and tumor levels. Plasma *CTSL* was below detectable limits in all but two samples; thus, no associations were observed. Plasma *CTSS* was not associated with tumor *CTSL* or *CTSS* expression (*p* > 0.300 for both). Decreases in body fat were associated with decreases in FFAs and IL-6. IL-6 was positively associated with other cytokines (hsCRP and TNFα).

**TABLE 1:** Correlations between changes in tumor proliferation rate and expression of *CTSL* and other biomarkers in prostate cancer patients participating in a randomized controlled weight loss trial.

	*CTSL*-mRNA	Change in lean mass (g)	Change in body fat (%)	Change in FFAs (mEq)	Change in insulin (mU/L)	Change in IL-6 (pg/ml)	Change in TNFα (pg/ml)	Change in hsCRP (mg/dl)
Change in Ki67 (% staining)								
ρ	0.304	−0.912*	−0.340	0.410	−0.650*^a^	−0.814*	0.419	−0.719*
*p*	0.393	0.001	0.370	0.273	0.042	0.014	0.301	0.045
*N*	10	9	9	9	10	8	8	8
*CTSL*-mRNA								
ρ		−0.391	−0.150	0.527	−0.643*	−0.103	0.600	0.164
*p*		0.235	0.659	0.096	0.024	0.777	0.067	0.651
*N*		11	11	11	12	10	10	10
Change in lean mass (g)								
ρ			0.248	−0.352	0.452*	0.331	0.015	0.203
*p*			0.195	0.072	0.014	0.106	0.942	0.330
*N*			29	27	29	25	25	25
Change in body fat (%)								
ρ				0.428*	0.432*	0.411*	0.069	0.277
*p*				0.026	0.019	0.041	0.742	0.180
*N*				27	29	25	25	25
Change in free fatty acids (mEq)								
ρ					−0.700*^b^	0.219	0.283	0.190
*p*					0.016	0.304	0.181	0.375
*N*					11	24	24	24
Change in insulin (mU/L)								
ρ						0.441*	0.181	0.183
*P*						0.024	0.377	0.371
*N*						26	26	26
Change in IL-6 (pg/ml)								
ρ							0.471*	0.716*
*P*							0.015	0.001
*N*							26	26
Change in TNFα (pg/ml)								
ρ								0.170
*p*								0.407
*N*								26

*CTSL*, Cathepsin-L; FFAs, free fatty acids; IL-6, Interleukin-6; TNFα, tumor necrosis factor-alpha; hsCRP, high-sensitivity C-reactive protein. *Uncorrected *p* < 0.05. ^*a*^For *n* = 20, ρ = −0.208, *p* = 0.380. ^*b*^For *n* = 28, ρ = 0.002, *p* = 0.991.

### Microbiota Associations

**FIGURE 2:**
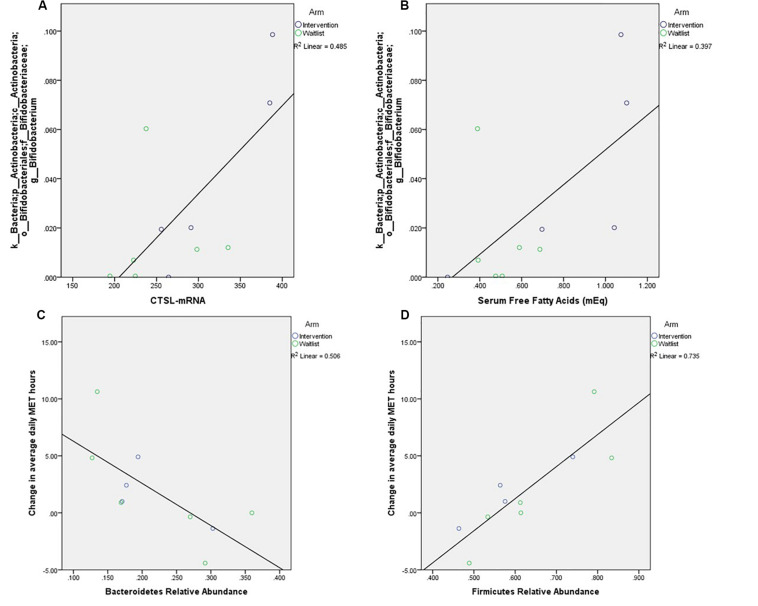
Relationships between relative abundance of the most abundant genus within these samples, Bifidobacterium, and tumor expression of *CTSL* (**A**, *p* = 0.039) and free fatty acids (**B**, *p* = 0.019), as well as correlations between change in self-reported weekly activity measured in average daily metabolic equivalent hours and the phyla **(C)** *p* = 0.019, **(D)** *p* = 0.003.

Prior to prostatectomy, Firmicutes (60.7 ± 12.9%) were the most abundant phyla, followed by Bacteroidetes (23.1 ± 8.5%), Proteobacteria (8.3 ± 6.4%), and Actinobacteria (6.4 ± 5.9%). The genus Bifidobacterium was positively correlated with *CTSL* (*p* = 0.039) and FFA (*p* = 0.019, Figure 2). The two most abundant bacterial phyla were associated with change in PA over the course of the study, with Bacteroidetes having an inverse correlation to change in MET hours (*p* = 0.021) and Firmicutes having a positive correlation to change in MET hours (*p* = 0.002). No measures of alpha diversity were associated with Ki67 or *CTSL* (all *p*-values greater than 0.500).

## Discussion

Given the unexpected results of our clinical trial that sought to slow tumor proliferation rate via negative energy balance, we conducted additional analyses to better understand the mechanisms underlying these results. The observed model of the relationship between the intentional rapid weight loss observed in our cancer patients and the undesired outcome of increased tumor proliferation is presented in Figure 1B. Rapid weight loss did not result in increased FFAs, possibly due to potential relative weight stabilization at the time of prostatectomy (20). Given that participants were on study 17–95 days, we did not assess absolute energy balance at the time of prostatectomy (6). No associations between FFAs and cytokines were observed, but cytokines trended together. The observation that IL-6 (as well as CRP and TNFα) decreased with loss of body fat was expected (21), but did not fit our proposed model in which body fat loss correlates with increased FFAs.

Increased FFAs were marginally associated with *CTSL* (*p* = 0.096) and loss of lean mass was associated with an increase in Ki67. The model was flawed in the assumption that *CTSL* and loss of lean mass would be highly correlated, which we did not observe. Larsson et al. (22) examined the effects of a lifestyle intervention program on circulating levels of *CTSL* and *CTSS* among healthy adults (*n* = 31, 29 females and 2 males). The intervention program resulted in modest weight reduction (mean decrease of 0.8 kg at 4 weeks and 1.4 kg at 8 weeks) and significant reductions of *CTSL* and *CTSS* levels in plasma after 4 and 8 weeks of intervention (22). Conversely, Naour et al. (23) reported an increase in *CTSL* (Pre = 2.14 ± 0.56; Post = 2.60 ± 0.75) among obese women (*n* = 29) who participated in a 6 months medically supervised caloric restriction, which resulted in a 5% reduction of BMI (Pre = 34.0 ± 0.69; Post = 32.1 ± 0.69). It is possible that we did not observe parallel results to previous studies given undetectable *CTSL* levels in most of our patients. Of note, our study is distinguished by inclusion of change in lean mass, which was not investigated in previous studies.

Our results show a remarkably strong relationship between loss of lean mass and Ki67. A plausible mechanism may be that the degradation of lean muscle into free amino acids supported tumor growth via mitochondrial, rather than glycolytic pathways (24). While the liver and kidneys tightly regulate the amino acid pool and nitrogenous waste products from deamination, the translocation of amino acids from muscle catabolism to rapidly proliferating tumor tissue is highly feasible (25). Emerging evidence supports the role of the L-Type Amino Acid Transporter (LAT-1) in increasing amino acid uptake to support proliferation in several cancer cell types (26). We queried our nanostring data to investigate this relationship, but gene expression of LAT-1 was unavailable. It is also noteworthy that upon enrollment in the study, these patients were sedentary and had increased BMI; given that fasting insulin levels were generally elevated, it is likely that underlying insulin resistance in skeletal muscle predisposed the tissue to sarcopenia (27), which may have been perpetuated by the diet-induced negative energy balance.

It is also important to note that this loss of lean muscle mass would be especially concerning for the proportion of patients for whom radiation and androgen deprivation therapy are required as adjuvant treatments that might further exacerbate muscle loss and quality of life. The loss of lean mass observed during the intervention may have potentially been prevented by an intervention that also included resistance training, which, in combination with aerobic training is most effective for improving outcomes in older adults (28). Indeed, resistance training would be expected to improve the hormonal and metabolic responses in the intervention (29), though it is unknown how this may have affected tumor gene expression. A recent meta-analysis of resistance exercise interventions in PCa patients observed improvements in body composition and lean body mass with several studies also reporting improvements in quality of life and depressive symptoms (30). Thus, resistance training may prove to be crucial for improving outcomes in PCa patients.

Most contrary to our hypothesis was that increased FFA would dysregulate glucose metabolism and increase tumor proliferation. Indeed, and perhaps most surprisingly, insulin was found to be the most strongly and inversely associated biomarker with Ki67, *CTSL*, and FFAs. It was previously observed that upregulation of *CTSL* would result in degradation of insulin receptors (31); however, our results suggest negative energy balance may have sensitized unaffected receptors and promoted normal glucose turnover and metabolism.

It has been suggested that the hyperinsulinemia of insulin resistance, or long-term adaptation to hyperinsulinemia, are means by which FFA release is downregulated (32). In our sample, decreased FFA was strongly correlated with increased insulin. Among obese women 6-month post bariatric surgery (Pre-surgical BMI: 41.1 ± 4.2; 6-month post-surgical BMI: 31.8 ± 4.2; weight loss: 26 ± 8 kg) decreased levels of plasma insulin and serum FFA, as well as a ∼50% increase in renal FFA uptake (which was well correlated with whole-body fat oxidation) have been reported (33). Among obese men with Type 2 diabetes, who underwent 10-week caloric restriction, a significant decrease in body weight, fat mass, and fat-free mass, as well as fasting glucose and insulin were observed. While not statistically significant (*p* = 0.11), basal FFA concentrations decreased after the diet (Pre = 679 ± 83; Post = 583 ± 120) (34).

A recent review of microbiota changes in non-exercise weight loss interventions in humans reported reductions in genera and species within Firmicutes phylum as well as decreases in the Firmicutes to Bacteroidetes ratio (35). Exercise has also been observed to be associated with changes in Firmicutes. Motiani et al. (36) found that among sedentary middle-aged insulin resistant males (*n* = 26) exercise training (6 sessions, 3× week over 2 weeks) decreased the ratio of Firmicutes/Bacteroidetes (time *p* = 0.04), mainly due to the increase in the relative abundance of Bacteroidetes phyla (time *p* = 0.03) while no change was found in the Firmicutes levels. Our study only reported post-intervention microbiota composition, so our results do not necessarily challenge previous findings. Our cross-sectional results regarding *CTSL* and microbes may be interesting as Bifidobacterium has been associated with obesity and weight loss in animals and humans (37, 38).

## Limitations and Conclusion

This exploratory study has generated several hypotheses related to weight loss and PCa; however, there are also several recognized limitations. First, we acknowledge that this was a secondary analysis of a clinical trial and was insufficiently powered to produce definitive results. The small sample sizes in various analyses may have generated false positives, so we were careful to not attribute clinical significance to low *p*-value findings. Additionally, objective and subjective data were both included in analyses; bias in self-reported PA and dietary recall data are common and could potentially distort findings.

Nonetheless, these results provide a revised framework for exploring the relationship between intentional weight loss, inflammation, and tumor proliferation. Future research should aim to determine if the inclusion of resistance training might moderate the loss of lean mass and undesirable changes in tumor biomarkers.

## Data Availability Statement

The datasets generated for this study are available on request to the corresponding author.

## Ethics Statement

The studies involving human participants were reviewed and approved by the University of Alabama at Birmingham Institutional Review Board. The patients/participants provided their written informed consent to participate in this study.

## Author Contributions

WD-W designed and obtained funding for the original study. WD-W and SR-B designed and obtained funding for this secondary analysis. AF conducted the analyses. AF, KS, and JB drafted the original manuscript. All authors provided critical feedback to the final manuscript.

## Conflict of Interest

The authors declare that the research was conducted in the absence of any commercial or financial relationships that could be construed as a potential conflict of interest.
